# Less is more: a rationalization of daily labwork

**Published:** 2018-11-12

**Authors:** Barry Chan, Alasdair Nazerali-Maitland, Wilma Hopman, David Zelt, Ross Morton

**Affiliations:** 1 Department of Internal Medicine, Queen’s University, Ontario, Canada; 2 Centre of Health Education Scholarship, Vancouver General Hospital, British Columbia, Canada; 3 Kingston General Hospital Clinical Research Centre, Ontario, Canada; 4 Department of Vascular Surgery, Kingston General Hospital, Ontario, Canada; 5 Department of Nephrology, Dalhousie University, Nova Scotia, Canada

## Introduction

Routine daily phlebotomy is often ordered for admitted patients after the established diagnostic algorithm and plan have been made and can often act as a replacement for direct patient care.^1^ The literature critiques gratuitously-ordered imaging studies, however a greater problem exists whether it is necessary for healthcare practitioners to order daily blood work to trend the progress of ongoing patient management.^2^ Measurable drawbacks are associated with excess laboratory test use and there are significant aspects of patient advocacy to consider: nursing resources spent on phlebotomy, risk of infection from an acquired superficial thrombophlebitis or cellulitis, the need for transfusion caused by the iatrogenic anemia from repeat phlebotomy, and in many cases, the perceived need for continuous intravenous access. It has been demonstrated that order-entry systems inquiry of the necessity of repeat laboratory testing can significantly decrease variability of test ordering.^3^ This study shows that providing Clinical Teaching Units (CTUs) with cost-analysis weekly, laboratory orders would decrease without compromising patient care through increasing residents’ knowledge of laboratory costs in Canada.

## Methods

Internal Medicine Residents were asked to estimate the costs incurred from phlebotomy related tests per patient per day (PPPD), which was done by completion of a survey distributed to key informants. Throughout the six-month study, four Internal Medicine CTUs were given weekly expenditure reports detailing their respective and average spending. Both in-hospital mortality rates and hospital readmission rates were tracked as surrogates of patient safety. The primary endpoint measured was laboratory test expenditure which reflected the number of total labs ordered. Time-series analysis, over the six-month period was performed to determine peak and trough spending.

## Results

**Figure 1:**
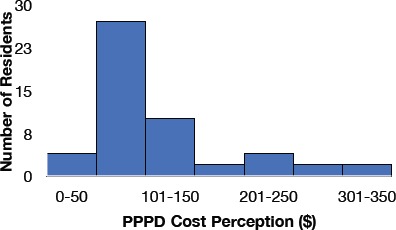
Histogram displaying residents’ knowledge, or perception of average PPPD expenditure on phlebotomy related tests.

**Figure 2:**
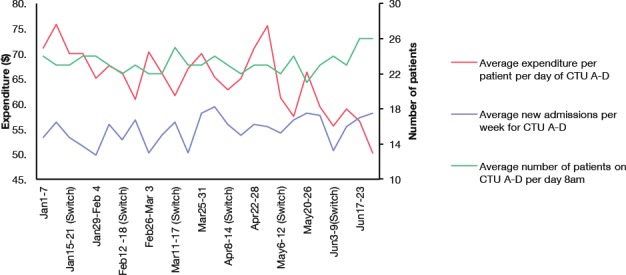
Average expenditure PPPD over time. *Total expenditure of all four teams on week 1 was $47,800.89 with an average of $71.13 PPPD. The total weekly expenditure maximum at week 2 was $48,842.00 with an average of $75.84 PPPD. The minimum occurred at week 26, being $36,534.00 with an average of $50.18 PPPD. Average spending over the 6-month period was $64.65 PPPD*.

Residents surveyed were unable to accurately estimate laboratory costs (Figure 1). Spending PPPD was reduced by 33% over six months with a maximum at $75.84PPPD with a minimum of $50.18PPPD (figure 2). Readmission rates through the study ranged from 6.7% to 14.5% (p<0.001). However, no change in mortality was observed (p=0.715).

## Discussion

This Canadian study shows that reductions in test ordering can be demonstrated by simply displaying this unique variable (PPPD) and comparing teams’ expenditure. These results support implementation of a “Choosing Wisely” curriculum to Canadian trainees as there is currently no mechanism to regulate investigation ordering.^2^

This study was limited to a single academic tertiary care centre, which constrains the generalizability of the findings. Due to the lack of a control group, there may be confounders such as the progression of an in-training-physician’s competency over time and seasonal disease variation. Increasing duration of data collection may reveal additional trends.

### Conclusion

Future studies can explore two programs of similar size in separate geographic locations (case versus control). This method might possess intrinsic biases, as residents may rotate through institutions monthly. By integrating financial stewardship and feedback into postgraduate medical education curricula, there is potential to facilitate the implementation of cost-conscious patient care as an essential component of the practice of medicine.
